# Different expression pattern of human cytomegalovirus-encoded microRNAs in circulation from virus latency to reactivation

**DOI:** 10.1186/s12967-020-02653-w

**Published:** 2020-12-09

**Authors:** Wanqing Zhou, Cheng Wang, Meng Ding, Yuying Bian, Yujie Zhong, Han Shen, Junjun Wang, Chen-Yu Zhang, Chunni Zhang

**Affiliations:** 1 Department of Laboratory Medicine, Nanjing Drum Tower Hospital, The Affiliated Hospital of Nanjing University Medical School, Nanjing University, Nanjing, 210008 China; 2 Department of Clinical Laboratory, Jinling Hospital, State Key Laboratory of Analytical Chemistry for Life Science, Nanjing University School of Medicine, Nanjing University, Nanjing, 210002 China; 3 Jiangsu Engineering Research Center for microRNA Biology and Biotechnology, Advance Research Institute of Life Sciences, School of Life Sciences, Nanjing University, Nanjing, 210046 China; 4 Department of Urology, Nanjing Drum Tower Hospital, The Affiliated Hospital of Nanjing University Medical School, Nanjing, 210008 China

**Keywords:** Human cytomegalovirus, Serum microRNA, HCMV DNA load, Latency, Reactivation, Switch

## Abstract

**Background:**

Human cytomegalovirus (HCMV) is a beta-hersvirinae that has a high latent infection rate worldwide and can cause serious consequences in immunocompromised patients when reactivation; however, the mechanism of how HCMV convert from latent to reactivation has rarely been investigated. In the present study, we aimed to perform a comprehensive analysis of the HCMV-encoded microRNA (miRNA) profile in serum of patients upon HCMV reactivation from latency and to further evaluate its clinical significance for the disease monitoring and preventing usefulness.

**Methods:**

Serum samples from 59 viremia patients and 60 age-gender matched controls were enrolled in this study for screening and validation of different expression of HCMV miRNAs. Serum concentrations of 22 known HCMV miRNAs were determined by a hydrolysis probe-based stem-loop quantitative reverse transcription polymerase chain reaction (RT-qPCR) assay. HCMV DNA was measured by quantitative real-time PCR (qPCR) with the whole blood sample. Serum HCMV IgG and IgM were assessed using enzyme linked immunosorbent assay (ELISA). Another 47 samples from 5 patients at different time points were collected to evaluate the monitoring effectiveness and disease prediction ability of differential expression HCMV-miRNAs during the antiviral treatment.

**Results:**

The RT-qPCR analysis revealed that the serum levels of 16 of the 22 examined HCMV miRNAs were elevated in HCMV viremia patients compared with controls, and a profile of 8 HCMV miRNAs including hcmv-miR-US25-2-3p, hcmv-miR-US4-5p, hcmv-miR-US25-2-5p, hcmv-miR-US25-1-3p, hcmv-miR-US25-1, hcmv-miR-UL36, hcmv-miR-UL148D, hcmv-miR-US29-3p were markedly elevated (fold change > 2, P < 0.01). Receiver operating characteristic curve (ROC) analysis were performed on the selected HCMV-miRNAs in all of the patients and controls that enrolled in this study, and which ranged from 0.72 to 0.80 in the autoimmune patients. In addition, hcmv-miR-US25-1-3p levels were significantly correlated with HCMV DNA load (r = 0.349, P = 0.007), and were obviously higher in the reactivation set than the latency set in the autoimmune patients, which could be a predictor for the monitoring of the antiviral treatment.

**Conclusions:**

HCMV miRNAs profile showed markedly shift-switch from latency to reactivation in circulation from HCMV infected patients and hcmv-miR-US25-1-3p may be served as a predictor for the switch upon reactivation from latency in patients suffered with autoimmune diseases.

## Background

Human cytomegalovirus (HCMV) is a member of the herpesviridae family, beta-hersvirinae subfamily that latently infects approximately 70–100% of the population worldwide in their lifetime [1]. Initial infection and reactivation of HCMV usually does not result in morbidity in healthy individuals, whereas reactivation of HCMV from the latency in immunocompromised people, such as AIDS patients, solid organ transplant recipients and neonates can lead to severe morbidity and mortality [2–4]. However, the mechanisms involving in HCMV latency and reactivation remain poorly understood.

MicroRNAs (miRNAs) are a subset of non-coding RNA molecules (19–23 nucleotides in length) that mediate post-transcriptional gene silencing. HCMV encodes at least 26 mature miRNAs, which were derived from 15 stem-loop precursors and have been implicated in the regulation of viral replication, immune modulation, and immune evasion [5, 6]. Differential HCMV encoded miRNAs expression was observed in the latency and activation infection by HCMV in vitro. For instance, hcmv-miR-UL148D facilities latent viral infection by modulating the IER5-CDC25B axis in host cells [7]. Hcmv-miR-UL112-1 can attenuate replication of HCMV and implicates in latency control of HCMV by targeting HCMV IE1, UL112/113, UL120/121 and UL144 [8, 9]. However, all of the HCMV miRNAs that discovered currently were detected in infected fibroblast cells [10, 11], which due to the lack of appropriate cell-lines or animal models for studying HCMV latency.

Circulating miRNAs could be novel biomarker for the diagnosis of virous diseases, including viral infection diseases [6, 12, 13]. In vivo evidence of the link between HCMV miRNAs and diseases processes is now emerging, with the description of hcmv-miR-UL112-3p as a biomarker of essential hypertension, diabetes mellitus and glioblastoma [14, 15], and hcmv-miR-UL22A-5p as a biomarker in solid organ transplantation [16]. Our group also demonstrated a distinct expression pattern of HCMV-encoded miRNAs in oral lichen planus (OLP) [17]. Moreover, one recent study showed that there was a different hcmv-miRNAs pattern between latent and lytic in vitro [11]. Nevertheless, no report about the relationship between the HCMV DNA load and HCMV miRNA’s expression levels in vivo. In addition, expression patterns of HCMV miRNAs and their roles in the transformation from latency to reactivation have not yet been examined in vivo.

Since understanding the HCMV miRNA expression pattern during latency phase, the reactivation phase and the shift expression between the above two phases will offer great benefit for HCMV associated diseases therapy, and may also provide clues for preventing reactivation of the virus from latency. In the present study, we assessed the in vivo expression pattern of HCMV miRNAs in the patients which with the detection of HCMV IgG seropositive, IgM seronegative, differ by the HCMV DNA level up and below for the 500 IU/mL (viremia or not which equivalent to reactivation or latency phase), and examined their potential as predictors of clinical and virological endpoints. We found a panel of HCMV-encoded miRNAs that showing different expression level between the latency and the reactivation, and some may be used as HCMV indicators, especially in the patients who suffered with autoimmune diseases and co-infected with the latency HCMV virus.

## Methods

### Participants and study design

**Fig. 1:**
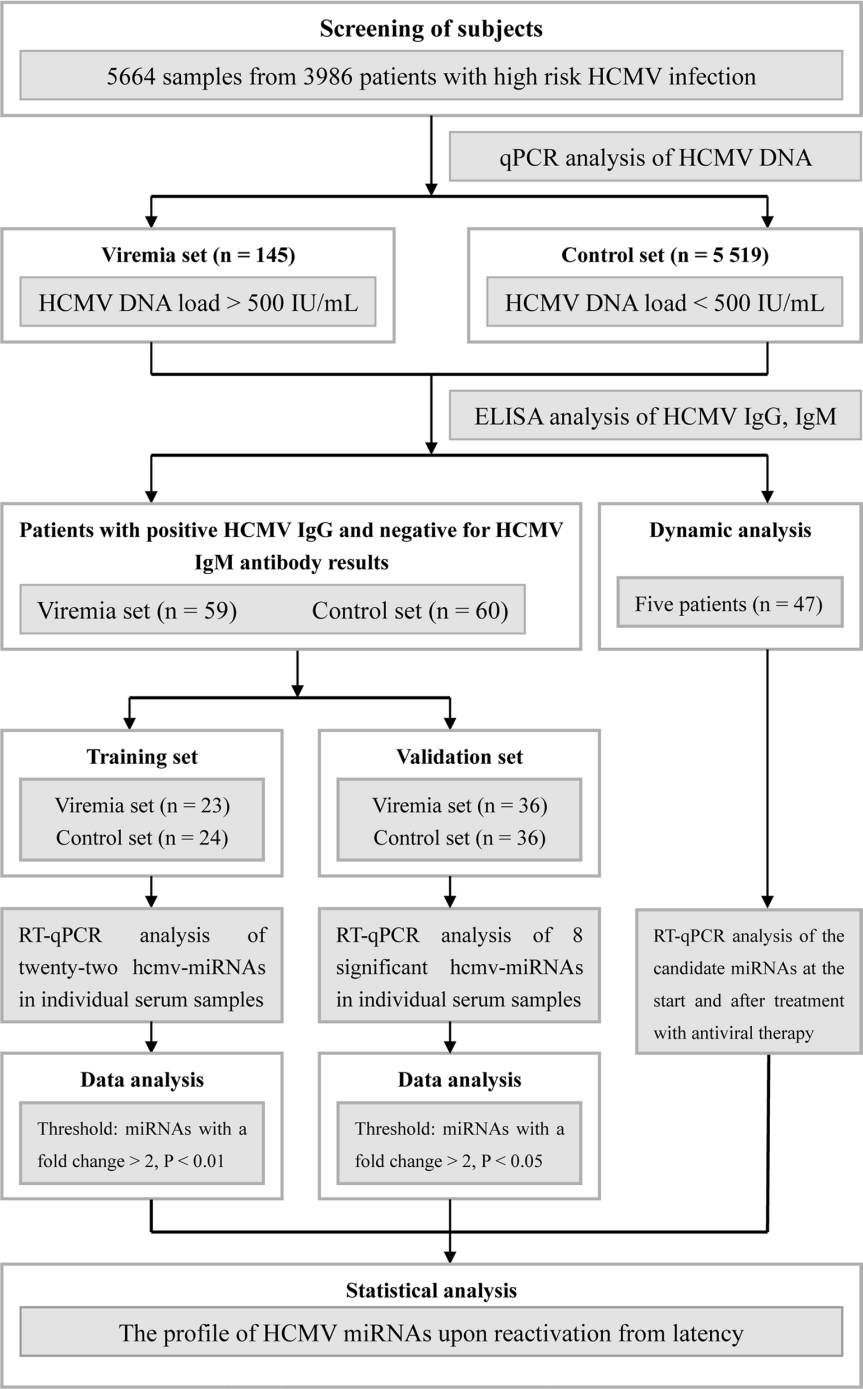
Overview of the experimental design

A total of 3986 subjects with high risk HCMV infection [2] mainly from the Departments of respiratory (27.2%), hematology (17.6%), immunology (13.6%), infection disease (12.9%), gastroenterology (8.8%), intensive care unit (3.0%) and others (16.9%) were recruited in this study. All the patients were hospitalized in Nanjing Drum Tower Hospital between January 2016 and March 2017. After HCMV DNA load and HCMV serological examination, a training cohort that containing 23 patients with HCMV viremia (HCMV DNA > 500 IU/mL), HCMV IgG seropositive and HCMV IgM seronegative as the case set (defined as reactivation infectious), and another 24 patients with HCMV DNA levels less than 500 IU/mL, HCMV IgG seropositive and HCMV IgM seronegative as the control set (defined as latency infectious) was used for screening the differential expression pattern of HCMV miRNAs. A validation cohort that containing 36 patients for the case set and 36 patients for the control set with the same above criterial was used to confirm the results of the training cohort. An additional independent cohort including 47 samples from 5 patients with leukemia (2 severe aplastic anemia patients, 1 myelodysplastic syndromes patient, 1 acute myeloid leukemia M2a patient and 1 acute lymphoblastic leukemia patient) who underwent bone marrow transplantation (the samples were collected at different time points during the antiviral therapy with ganciclovir) were also collected. The overall study design is shown in Fig. 1. All clinical data and blood samples were obtained from participants who had given written informed consent, according to protocols approved by the Ethics Committee of Nanjing Drum Tower Hospital. For all the patients, the age, gender, diagnosis, white blood cell count, C reactive protein (CRP), HCV, EBV and HIV status were recorded and used for the study (Table 1).

**Table 1:** Demographic and clinical features of training set and validation set in the present study

Variable		Training set			Validation set		
		Viremia (n = 23)	Control (n = 24)	P values	Viremia (n = 36)	Control (n = 36)	P values
Age, years^a^	44.1 ± 4.0		51.9 ± 3.6	0.15^‡^	52.8 ± 3.3	49.7 ± 3.2	0.50^‡^
Sex, n				0.19^§^			0.48^§^
Male	9 (39.1%)		14 (58.3%)		19 (52.8%)	16 (44.4%)	
Female	14 (60.9%)		10 (41.7%)		17 (47.2%)	20 (55.6%)	
HCMV DNA Level^b^	1920 (1100, 3680)		–		1995 (795.5, 9995)	–	
Anti-HCMV IgG (IU/mL)^a^	1.59 ± 0.16		1.74 ± 0.19	0.57 ^‡^	1.94 ± 0.16	1.63 ± 0.15	0.17^‡^
Anti-HCMV IgM, n							
Positive	0 (0%)		0 (0%)		0 (0%)	0 (0%)	
Negative	23 (100%)		24 (100%)		36 (100%)	36 (100%)	
HCV, n							
Positive	0 (0%)		0 (0%)		0 (0%)	0 (0%)	
Negative	23 (100%)		24 (100%)		36 (100%)	36 (100%)	
EB, n				0.19^§^			0.47^§^
Positive	11 (47.8%)		16 (66.7%)		13 (36.1%)	16 (44.4%)	
Negative	12 (52.2%)		8 (33.3%)		23 (63.9%)	20 (55.6%)	
HIV, n							
Positive	0 (0%)		0 (0%)		0 (0%)	0 (0%)	
Negative	23 (100%)		24 (100%)		36 (100%)	36 (100%)	
Leukemia, n				0.61^§^			1^§^
Yes	7 (30.4%)		9 (37.5%)		13 (36.1%)	13 (36.1%)	
No	16 (69.6%)		15 (62.5%)		23 (63.9%)	23 (63.9%)	
Autoimmune diseases, n				0.81^§^			0.44^§^
Yes	6 (26.1%)		7 (29.2%)		9 (25%)	12 (33.3%)	
No	17 (73.9%)		17 (70.8%)		27 (75%)	24 (66.7%)	

^‡^Student *t* test

^§^Two-sided χ^2^ test

^a^Age data and anti-HCMV IgG are presented as the mean ± SD

^b^HCMV DNA level are presented as median (25% Percentile, 75% Percentile)

### HCMV DNA load

The HCMV DNA level in peripheral blood leukocytes were determined by quantitative real-time PCR (qPCR). In brief, DNA was extracted from 200 µL peripheral blood leukocytes using QIAamp DNA Mini kit (Qiagen, Hilden, Germany) according to the manufacturer's protocol. Two microliters of DNA were tested with TaqMan PCR assays using diagnostic kit for quantification of human cytomegalovirus DNA (DAAN GENE, Guangzhou, China) on a Roche LightCycler^®^ 96 PCR System (Roche diagnostics, Mannheim, Germany) according to the manufacturer's protocol. Ten-fold diluted recombinant plasmid that contained the HCMV target sequence was used to construct calibration curve. The absolute HCMV DNA level of each sample were calculated through the calibrator. Results were expressed as IU per 1 mL blood [18].

### Anti-HCMV IgG and IgM antibodies determination

Serum anti-HCMV IgG and IgM were tested using a commercially available ELISA kit (MEDSON, NJ, USA) according to the manufacturer’s instructions. In brief, serum (1:100) was added to the 96-well plate which containing HCMV antigen and incubated at 37 ℃ for 1 h, the mixture was then washed for four times and incubated with a horse-radish peroxidase conjugated at 37 °C for 1 h. After four times washing, reactivity was determined using o-phenylenediamine and the reaction was blocked with 2.5 M sulfuric acid. For the IgG-ELISA, a calibration curve, calibrated against the 1st WHO international standard, was used to quantitatively determine IgG antibody concentrations in each sample. For the IgM-ELISA, the test results were calculated using the optical density (OD) value at 450 nm, and the cut-off value for positivity was OD > 0.25.

### Serum RNA isolation and RT-qPCR assay

Total RNA was extracted from 100 μL of serum using a 1-step phenol/chloroform purification method and precipitated using isopropyl alcohol as previously described [17]. In brief, 100 μL of serum was mixed with 300 μL deionized water, 200 μL acid phenol, and 200 μL chloroform. The mixture was vortex-mixed vigorously and incubated at room temperature for 15 min. After phase separation, the aqueous layer was mixed with 1.5 volumes of isopropyl alcohol and 0.1 volumes of 3 mol/L sodium acetate (pH 5.3). The solution was stored at – 20 °C for 1 h. The RNA pellet was obtained by centrifugation at 16,000*g* for 20 min at 4 °C. The resulting RNA pellet was washed once with 750 mL/L ethanol and dried for 10 min at room temperature. The pellet was dissolved in 20 μL of RNase-free water and stored at − 80 °C. To control the variability in RNA extraction and purification procedures, an exogenous plant small molecular RNA named MIR2911 (5′-GGCCGGGGGACGGGCUGGGA-3′), was spiked into each sample with a final concentration of 10^6^ fmol/L during RNA isolation as a synthetic external reference for the normalization of serum miRNAs [19]. Hydrolysis probe-based quantitative reverse transcription polymerase chain reaction (RT-qPCR) was carried out using a TaqMan miRNA PCR kit (Applied Biosystems, Foster City, CA, USA) according to the manufacturer’s instructions with a minor modification as previously described [19]. Briefly, 2 μL of total RNA was reverse transcribed to cDNA using AMV reverse transcriptase (TaKaRa, Dalian, China) and the stem-loop RT primer (Applied Biosystems, Foster City, CA, USA). Real-time PCR was performed using hydrolysis miRNA probes on a Roche LightCycler^®^ 96 PCR System (Roche diagnostics, Mannheim, Germany). All reactions, including no-template controls, were performed in triplicate. The Cq values were determined using the fixed threshold settings. Relative levels of HCMV miRNAs were then normalized to exogenous MIR2911 and were calculated using comparative Cq method (2^−ΔCq^).

### Statistical analysis

Statistical analysis was performed with GraphPad Prism 6.0 software or SPSS statistical software (version 16.0). The miRNA concentrations were represented as means and standard errors (Mean ± SEM) and other clinical variables were showed as Mean ± SD or Median (interquartile range). The data analyses were performed using the non-parametric Mann–Whitney tests, Chi-square test and Pearson correlation analyses. Statistically significant was defined as a P < 0.05. For each miRNA, a receiver operating characteristic (ROC) curve was generated. The area under curve (AUC) values and 95% confidence interval (CI) were calculated to determine the specificity and sensitivity of diagnosis of HCMV reactivation.

## Results

### HCMV DNA viral load and serological results

We examined the HCMV viral load in 5664 samples of peripheral blood leukocytes that collected from 3986 patients by quantitative real-time PCR. Among which 145 whole blood samples were defined as viremia with the HCMV DNA level upper than 500 IU/mL and others with HCMV DNA level lower than 500 IU/mL. Of the 145 whole blood samples, 59 non-repetitive patients with HCMV-IgG seropositive, HCMV-IgM seronegative were enrolled in this study. In the meanwhile, another 60 patients with HCMV-IgG seropositive, HCMV-IgM seronegative and HCMV DNA level below 500 IU/mL were selected as controls (Fig. 1). The mean levels of HCMV DNA viral load for the 59 viremia patients were 1930 (853, 6810) IU/mL, 1920 (1100, 3680) IU/mL in the training cohort and 1995 (795.5, 9995) IU/mL in the validation cohort, respectively (Table 1). For the concentrations of anti-HCMV IgG, there was no significant difference between the HCMV viremia patients (n = 59) and the controls (n = 60) (t = 0.7794, P = 0.4373). Similar results were also observed in the training cohort and the validation cohort (t = 0.5766, P = 0.5671 and t = 1.384, P = 0.1709), respectively.

### Expression profiles of HCMV-encoded miRNAs by RT-qPCR analysis

In the training cohort, 22 HCMV-encoded miRNAs (http://www.mirbase.org) were measured using RT-qPCR assay in individual serum samples from 23 patients with a HCMV DNA > 500 IU/mL (referred as case set) and 24 patients with a HCMV DNA < 500 IU/mL (referred as control set) (Fig. 1). Sixteen of the 22 HCMV-encoded miRNAs were up-regulated in case set when compared with control set (P < 0.05), among which 8 miRNAs were significantly up-regulated with a fold change > 2, and P < 0.01, including hcmv-miR-US25-2-3p, hcmv-miR-US4-5p, hcmv-miR-US25-2-5p, hcmv-miR-US25-1-3p, hcmv-miR-US25-1, hcmv-miR-UL36, hcmv-miR-UL148D and hcmv-miR-US29-3p (Table 2).

**Table 2:** Expression profile of HCMV-encoded miRNAs in the training set

HCMV encoded miRNAs	Viremia (n = 23)	Control (n = 24)	Fold change	P values^†^
US25-2-3p	6.41 ± 2.07 × 10^–2^	2.66 ± 0.57 × 10^–2^	2.43	0.0028
US4-5p	3.51 ± 0.92 × 10^–3^	1.24 ± 0.31 × 10^–3^	2.90	0.0028
US25-2-5p	6.38 ± 1.69 × 10^–2^	2.38 ± 0.58 × 10^–2^	2.74	0.0034
US25-1-3p	1.98 ± 0.75 × 10^–2^	6.71 ± 1.59 × 10^–3^	2.95	0.0039
US25-1	2.46 ± 0.67 × 10^–3^	9.53 ± 2.28 × 10^–4^	2.62	0.0050
UL36	2.19 ± 0.55 × 10^–2^	9.97 ± 2.90 × 10^–3^	2.26	0.0055
UL148D	1.96 ± 0.50 × 10^–1^	6.98 ± 1.35 × 10^–2^	2.87	0.0055
US29-3p	2.00 ± 0.57 × 10^–3^	7.51 ± 1.67 × 10^–4^	2.72	0.0091
UL69	1.34 ± 0.35 × 10^–3^	6.09 ± 1.46 × 10^–4^	2.24	0.0117
US5-1	1.69 ± 0.53 × 10^–2^	8.74 ± 2.54 × 10^–3^	1.96	0.0117
US22-5p	2.01 ± 0.66 × 10^–1^	8.19 ± 1.80 × 10^–2^	2.51	0.0132
US33-3p	2.29 ± 0.52 × 10^–3^	1.07 ± 0.25 × 10^–3^	2.18	0.0132
UL22a	1.12 ± 0.29 × 10^–2^	4.79 ± 1.07 × 10^–3^	2.38	0.0171
UL70-5p	1.21 ± 0.36 × 10^–2^	5.29 ± 1.71 × 10^–3^	2.37	0.0262
UL36*	2.41 ± 0.76 × 10^–3^	6.76 ± 1.79 × 10^–4^	2.94	0.0276
US22-3p	4.38 ± 1.29 × 10^–3^	2.24 ± 0.62 × 10^–3^	1.97	0.0380
US5-2-3p	4.02 ± 1.13 × 10^–4^	1.36 ± 0.35 × 10^–4^	2.46	0.0721
UL112	3.00 ± 0.86 × 10^–2^	1.23 ± 0.34 × 10^–2^	2.09	0.0948
UL22a*	4.32 ± 1.29 × 10^–3^	1.53 ± 0.44 × 10^–3^	2.54	0.1178
UL112-5p	4.67 ± 1.36 × 10^–3^	1.95 ± 0.54 × 10^–3^	2.03	0.1281
US4-3p	6.24 ± 2.05 × 10^–3^	4.54 ± 1.87 × 10^–3^	1.23	0.2593
UL59	2.55 ± 0.64 × 10^–2^	1.40 ± 0.35 × 10^–2^	1.71	0.3546

miRNAs are presented as mean ± SEM

^†^ Mann–Whitney U test

### Confirmation of the up-regulated HCMV-encoded miRNAs

**Fig. 2:**
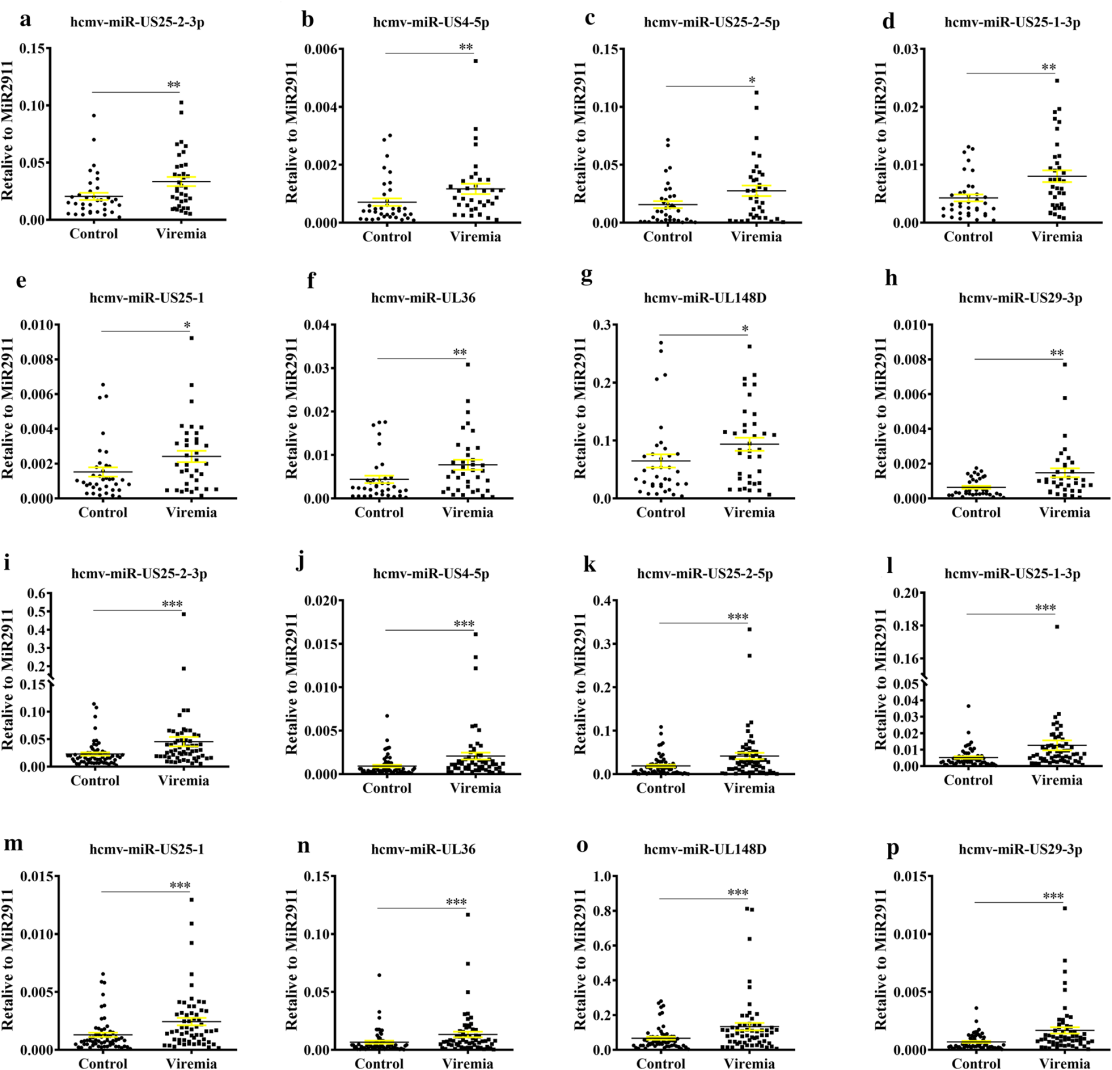
Serum levels of the 8 upregulated hcmv-miRNAs in the HCMV viremia patients as compared with control group in the validation set (**a**–**h**) and the combination set (**i**–**p**). **a**, **i** hcmv-miR-US25-2-3p; **b**, **j** hcmv-miR-US4-5p; **c**, **k** hcmv-miR-US25-2-5p; **d, l** hcmv-miR-US25-1-3p; **e, m** hcmv-miR-US25-1; **f, n** hcmv-miR-UL36; **g, o** hcmv-miR-UL148D; **h, p** hcmv-miR-US29-3p. Cq values were converted to relative concentrations normalized to MIR2911 values, and were calculated using the comparative Cq method (2^−ΔCq^). Each point represents the mean of triplicate sample. ^✻^, indicates that compared with the control group, P < 0.05; ^✻✻^,indicates that compared with the control group, P < 0.01; ^✻✻✻^, indicates that compared with the control group, P < 0.001. P‐values were measured by Mann–Whitney independent t test

Subsequently, the 8 up-regulated HCMV-encoded miRNAs were confirmed in an additional cohort including 36 cases and 36 controls (refereed as validation cohort). The 8 miRNAs exhibited consistent alterations as the results from the training cohort (Fig. 2a–h). Moreover, when combined the results of the training set and validation set (Fig. 2i–p), consistent with our expectations, the concentrations of all the eight hcmv-miRNAs were significantly increased in the viremia patients as compared with control group.

### HCMV miRNAs in the autoimmune disease patients

**Fig. 3:**
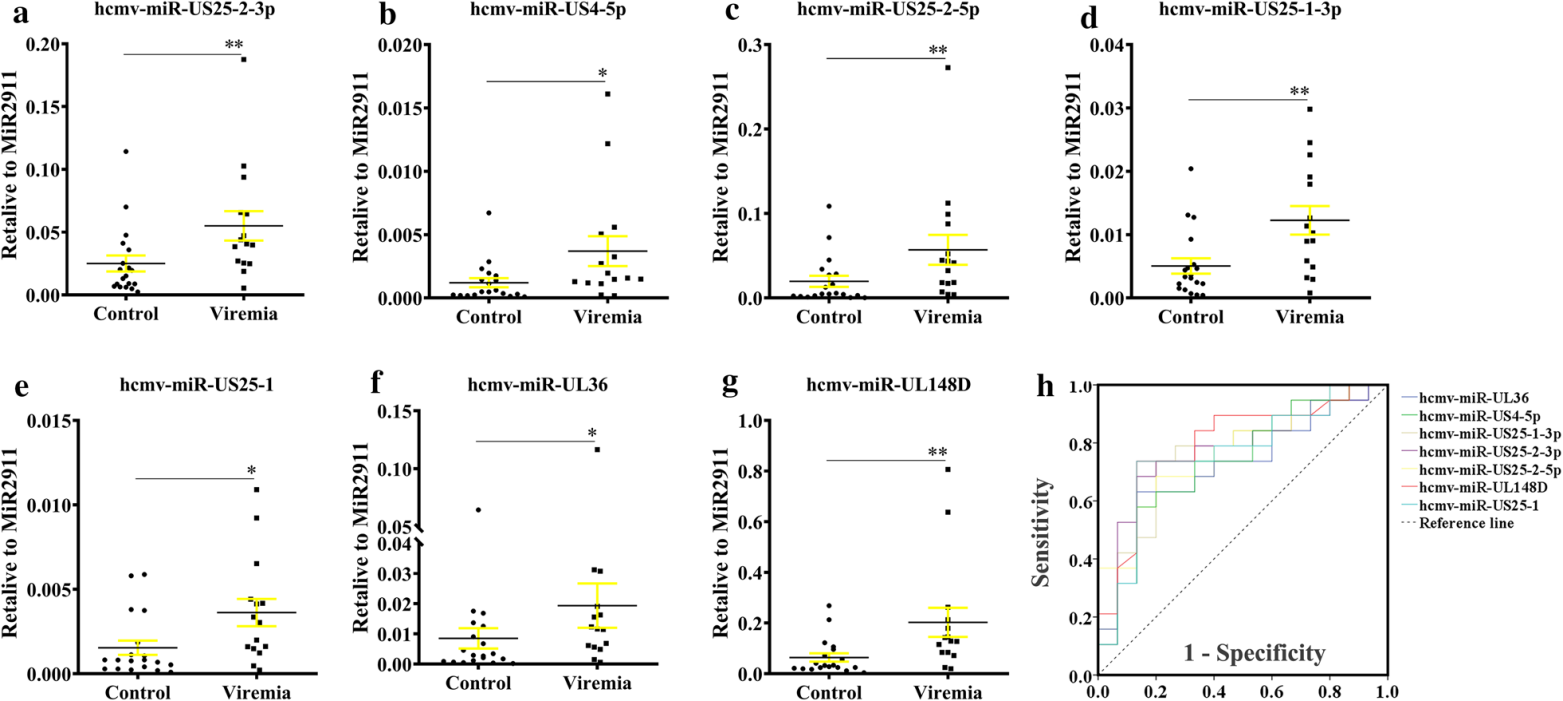
Serum levels and ROC curve analyzes of the 7 upregulated hcmv-miRNAs in the autoimmune disease patient subgroup. **a** hcmv-miR-US25-2-3p; **b** hcmv-miR-US4-5p; **c** hcmv-miR-US25-2-5p; **d** hcmv-miR-US25-1-3p; **e** hcmv-miR-US25-1; **f** hcmv-miR-UL36; **g** hcmv-miR-UL148D; **h** ROC analysis of the 7 upregulated hcmv-miRNAs in the autoimmune disease patients. Areas under ROC curve (AUCs) ranged from 0.72 to 0.80. Cq values were converted to relative concentrations normalized to MIR2911 values and were calculated using the comparative Cq method (2^−ΔCq^). Each point represents the mean of triplicate sample. ^✻^, indicates that compared with the control group, P < 0.05; ^✻✻^, indicates that compared with the control group, P < 0.01

We next analyzed the 8 up-regulated hcmv-miRNAs in the autoimmune disease patient subgroup, and found that seven hcmv-miRNAs were significantly up-regulated except for hcmv-miR-US29-3p. Notably, four miRNAs including hcmv-miR-US25-2-3p, hcmv-miR-US25-2-5p, hcmv-miR-US25-1-3p and hcmv-miR-UL148D were markedly increased with a P value of < 0.01 (Fig. 3a–g). Receiver operating characteristic curve (ROC) analysis on the seven selected hcmv-miRNAs yielded areas under ROC curve (AUCs) ranged from 0.72 to 0.80 (Fig. 3h). Using the optimal cutoff value, we obtained the following sensitivity and specificity values: hcmv-miR-US25-2-3p (AUC: 0.768, sen: 86.7%, spe: 68.4%, 95% CI 0.602, 0.935), hcmv-miR-US4-5p (AUC: 0.737, sen: 86.7%, spe:57.9%, 95% CI 0.565, 0.908), hcmv-miR-US25-2-5p (AUC: 0.765, sen: 80.0%, spe: 68.4%, 95% CI 0.605, 0.925), hcmv-miR-US25-1-3p (AUC: 0.772, sen: 80.0%, spe: 73.7%, 95% CI 0.610, 0.934), hcmv-miR-US25-1 (AUC: 0.761, sen: 86.7%, spe: 73.7%, 95% CI 0.592, 0.931), hcmv-miR-UL36 (AUC: 0.719, sen: 86.7%, spe: 63.2%, 95% CI 0.544, 0.894) and hcmv-miR-UL148D (AUC: 0.800, sen: 86.7%, spe: 73.7%, 95% CI 0.645, 0.955).

### Viral miRNAs expression pattern in the blood of patients with HCMV disease

**Fig. 4:**
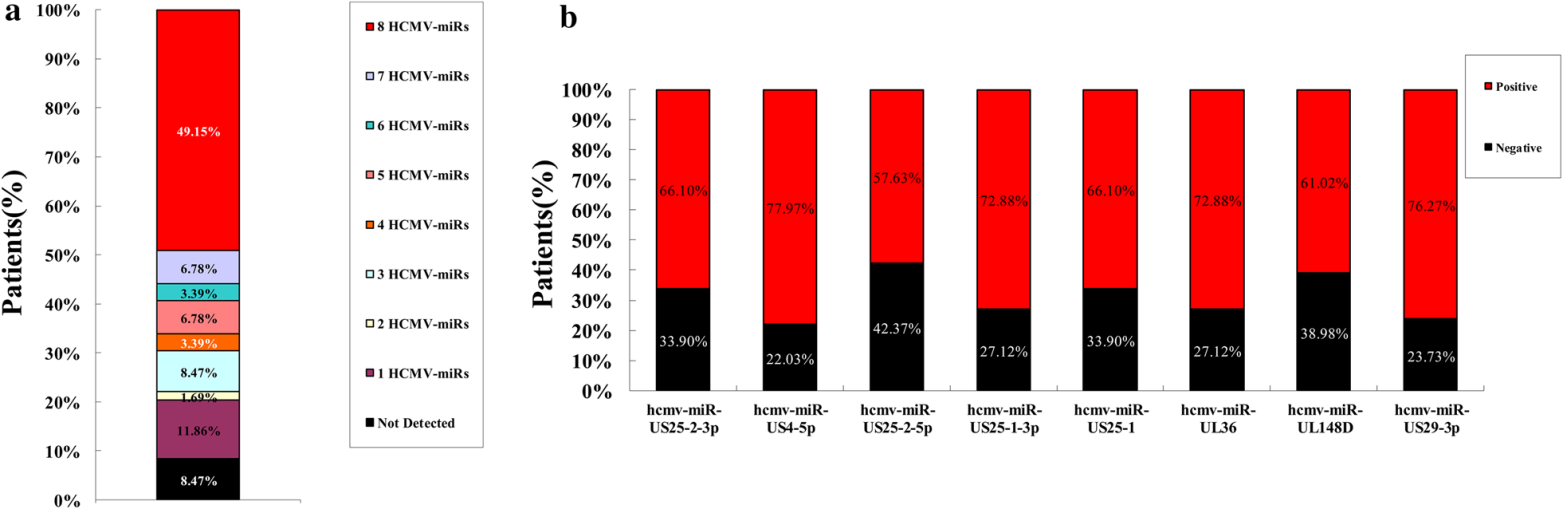
Hcmv-miRNAs as serum biomarkers for HCMV infection. **a** Percentage of patients positive for the serum expression of undetermined, one, two, three, four, five, six, seven or eight hcmv-miRNAs (hcmv-miR-UL36, hcmv-miR-US4-5p, hcmv-miR-US25-1-3p, hcmv-miR-US25-2-3p, hcmv-miR-US29-3p, hcmv-miR-US25-2-5p, hcmv-miR-UL148D and hcmv-miR-US25-1); **b** Percentage of patients positive for each of the serum hcmv-miRNAs (hcmv-miR-UL36, hcmv-miR-US4-5p, hcmv-miR-US25-1-3p, hcmv-miR-US25-2-3p, hcmv-miR-US29-3p, hcmv-miR-US25-2-5p, hcmv-miR-UL148D and hcmv-miR-US25-1)

In combined samples of the training set and validation set of patients (n = 59), we found that 91.53% patients had detectable expression of at least one hcmv-miRNA (11.86% with only one, 1.69% with two, 8.47% with three, 3.39% with four, 6.78% with five, 3.39% with six, 6.78% with seven and 49.15% with eight) (Fig. 4a). Analysis of individual hcmv-miRNA showed that hcmv-miR-US4-5p was the most commonly detected in 77.97% of the patient, followed by hcmv-miR-US29-3p with 76.27%, hcmv-miR-UL36 and hcmv-miR-US25-1-3p with 72.88%, hcmv-miR-US25-2-3p and hcmv-miR-US25-1 with 66.10%, hcmv-miR-UL148D with 61.02%, hcmv-miR-US25-2-5p with 57.63% (Fig. 4b).

### Association of serum hcmv-miRNAs’ levels with clinical parameters

**Fig. 5:**
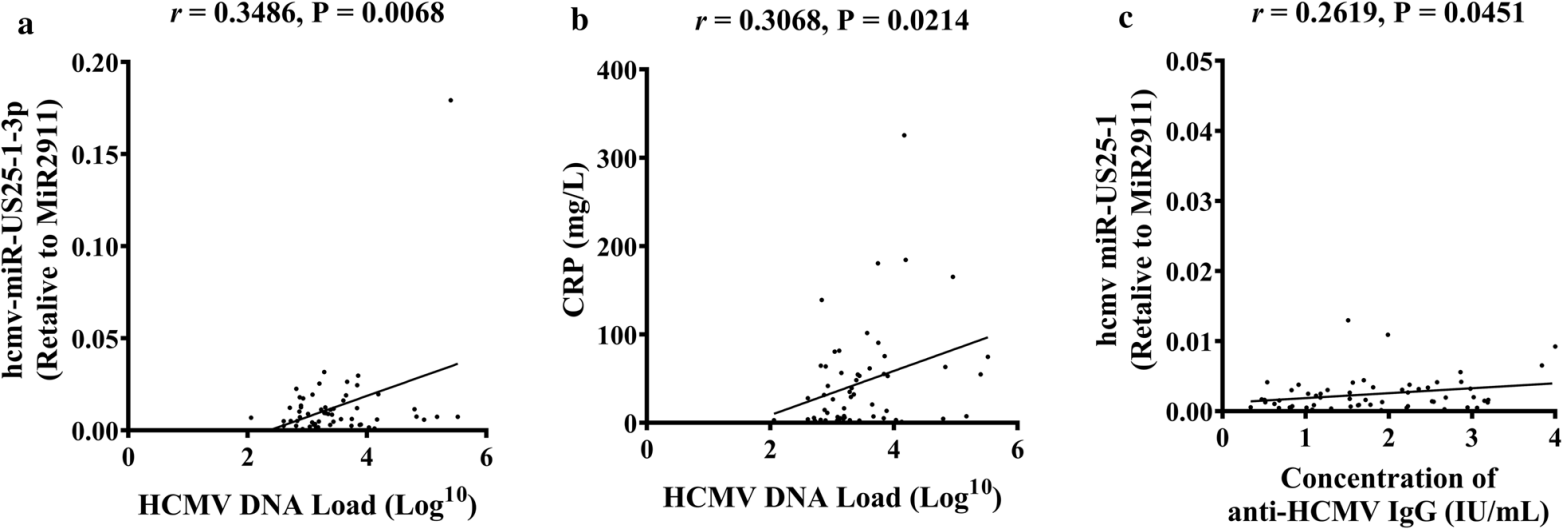
Associations of serum HCMV-miRNAs’ levels with clinical parameters. **a** Pearson correlation analysis for HCMV DNA levels and hcmv-miR-US25-1-3p; **b** Pearson correlation analysis for HCMV DNA and CRP; **c** Pearson correlation analysis for concentration of anti-HCMV IgG and hcmv-miR-US25-1

We subsequently wonder whether serum hcmv-miRNAs’ levels were correlated with clinical parameters. We evaluated the associations between the clinical features and miRNA abundance using Pearson correlation analysis in all of the studied individuals. Hcmv-miR-US25-1-3p levels were significantly correlated with HCMV DNA loads (r = 0.3486, P = 0.0068) (Fig. 5a). HCMV DNA levels was significantly correlated with CRP (r = 0.3068, P = 0.0214) (Fig. 5b), but not with WBC count (r = 0.0325, P = 0.80). The concentrations of anti-HCMV IgG was significantly correlated with hcmv-miR-US25-1 (r = 0.2619, P = 0.0451) (Fig. 5c) but not with other miRNAs. There was no significant difference in WBC count (t = 0.9736, P = 0.3323), CRP (t = 0.6670, P = 0.5062), PCT (t = 1.246, P = 0.2184), and ESR (t = 0.6731, P = 0.4242) between the viremia and the control group.

### Independent cohorts of antiviral treatment

**Fig. 6:**
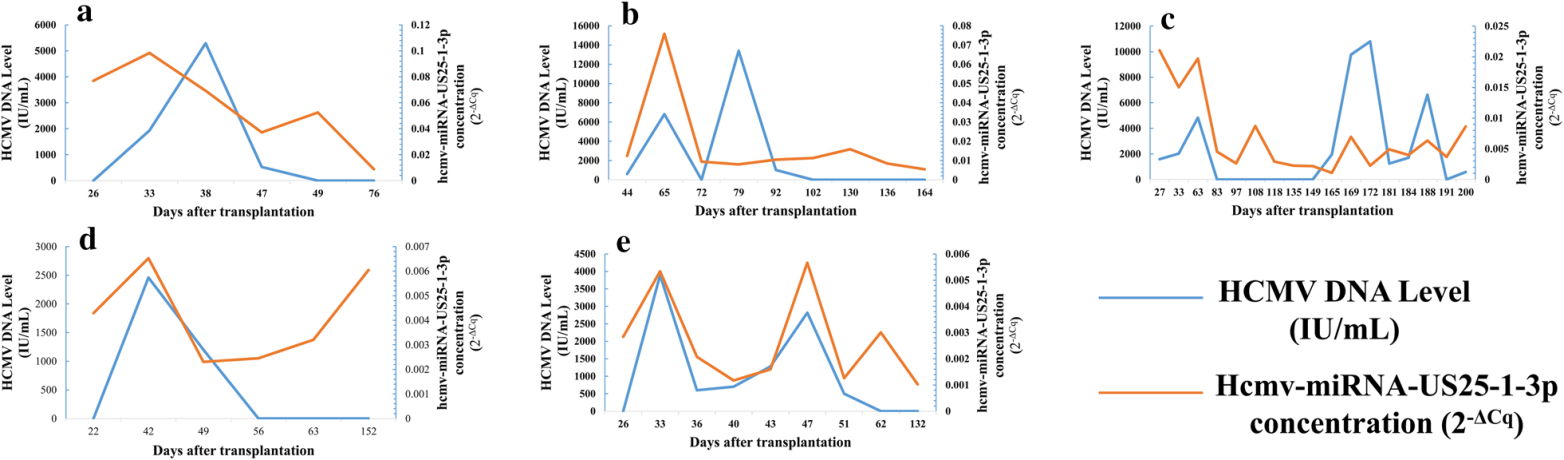
Dynamic changes of HCMV DNA and hcmv-miR-US25-1-3p during the treatment of anti-HCMV virus in five patients after bone marrow transplantation. **a** Patient No. 1 suffered with severe aplastic anemia with occurrence of viremia on the 33rd day after transplantation; **b** Patient No. 2 suffered with severe aplastic anemia with occurrence of viremia on 65th day after transplantation; **c** Patient No. 3 suffered with AML M2a with occurrence of viremia on 27th day after transplantation; **d** Patient No. 4 suffered with myelodysplastic syndromes with occurrence of viremia on 42nd day after transplantation; **e** Patient No. 5 suffered with ALM with occurrence of viremia on 33rd day after transplantation. The time of the patient underwent bone marrow transplant was taken as a starting monitor point (zero day) and the abscissa axis is based on the number of days after transplantation. The ordinates represented the levels of HCMV DNA and hcmv-miR-US25-1-3p, with blue line represented HCMV DNA level, and the red line represents hcmv-miR-US25-1-3p

Five patients were received antiviral therapy, including 2 patients with severe aplastic anemia, one with myelodysplastic syndromes, one with acute myeloid leukemia M2a and one with acute lymphoblastic leukemia respectively, who were underwent bone marrow transplantation. Forty-seven serum samples were collected at different time points during the antiviral therapy, and hcmv-miR-US25-1-3p levels was significantly correlated with HCMV DNA levels during the antiviral therapy (Fig. 6a–e).

The time of occurrence of viremia in the five patients was on the 33rd day (Fig. 6a), 65th day (Fig. 6b), 27th day (Fig. 6c), 42nd day (Fig. 6d), and 33rd day (Fig. 6e) after bone marrow transplantation, respectively. Of the five patients, the No. 3 patient developed viremia on 27th day (Fig. 6c) after allogeneic stem cell transplant, both HCMV DNA and hcmv-miR-US25-1-3p were at high levels at this time and decreased upon antiviral therapy. On 130th day after transplantation, the patient’s hematopoietic disease relapsed and worsened again, and was transferred to the ICU for emergency treatment. In the late phase of monitoring, the viral DNA showed a certain degree of volatility, while the expression of hcmv-miR-US25-1-3p level does not rise significantly. The No. 4 Patient developed viremia on day 42 (Fig. 6d) after transplantation. HCMV DNA levels was consistent with antiviral therapy, while hcmv-miR-US25-1-3p expressed earlier than the expression of HCMV DNA and decreased later than HCMV DNA during the antiviral therapy. Dynamic changes of HCMV DNA and hcmv-US25-1-3p levels in the 5 patients during antiviral therapy was showed in Additional file 1: Table S1.

## Discussion

Understanding HCMV shift from latency to reactivation offers great potential for therapy, as it may prevent reactivation of the virus from latency. At present, the majority of studies investigating the expression of HCMV-encoded miRNAs during latency in HCMV infected THP-1 cells at different infection stages, such as lytic and latent infection, and differential expression pattern of HCMV miRNAs may served as a feature of stage changes. In additional, dysregulated HCMV miRNAs also play important roles in regulating the expression of latent and lytic-related genes [20]. However, HCMV reactivation in those in vitro cell models is very ineffective, and may not accurately represent the natural latent infection [21]. Till date, there were only few reports about the HCMV infection in the peripheral blood mononuclear cells that were isolated from HCMV-IgG positive donor [20, 22]. Thus, the present study is aim to explore the expression characteristic of hcmv-miRNAs in the process of HCMV infection from latency to reactivation in vivo, and also to uncover the possible significance of specific hcmv-miRNAs in such process of viral infection. We analyzed the difference of HCMV-encoded-miRNAs expression pattern in the circulation of clinical patients who suffered with HCMV latency and reactivation. We found that 16 of 22 HCMV-encoded-miRNAs showed upregulation in the reactivation patients as compared with the latent controls. Among which, 8 hcmv-miRNAs, including hcmv-miR-US25-2-3p, hcmv-miR-US4-5p, hcmv-miR-US25-2-5p, hcmv-miR-US25-1-3p, hcmv-miR-US25-1, hcmv-miR-UL36, hcmv-miR-UL148D and hcmv-miR-US29-3p were steadily and markedly up-regulated in viremia group. These results indicate that the expression of HCMV miRNAs are strongly induced, and which are similar with previous studies that assessing hcmv-miRNAs expression in HCMV lytic infection models in vitro [10], as well as in solid organ transplant recipients in vivo [16].

The eight markedly upregulated HCMV miRNAs that were identified in our present study were also reported to be dysregulated in many other reports. For instance, a subset of HCMV-encoded-miRNAs showed remarkable increase upon reactivation of lytic infection, including hcmv-miR-US25-2-5p, hcmv-miR-US25-1-5p [20]. Of these miRNAs, hcmv-miR-US25-1, or hcmv-miR-US25-1-5p, is involved in diverse process, such as cell cycle control, virus replication inhibition, and apoptosis regulation [5, 6, 23]. Hcmv-miR-US25-1 was also demonstrated to inhibit HCMV DNA replication through the reduction of IE72 and pp65 expression [24], whereas hcmv-miR-US25-1-3p has the ability to down regulate CDK6 gene associated with suppress cell cycle progression [22, 25]. Notably, hcmv-miR-US25-2 can reduce HCMV replication by targeting not only the above targets but also eukaryotic translation initiation factor 4A1 (eIF4A1) [26]. Based on those results, we found that the upregulation of hcmv-miR-US25-1, hcmv-miR-US25-1-3p, hcmv-miR-US25-2-3p, and hcmv-miR-US25-2-5p upon the reactivation of HCMV may also involved in replication inhibition. Similarly, hcmv-miR-UL148D, which was robustly accumulated during the late stages of latent infection in host cells and facilities latent viral infection by modulating the IER5-CDC25B axis [7], was also increased upon reactivation in our study. For hcmv-miR-UL36, previous study revealed both strands of this miRNA were highly expressed upon reactivation from latency [20], and was similar with our in vivo results, which indicate that this miRNA was also participated in HCMV reactivation.

On the other hand, the hcmv-miRNA expression pattern in vitro was not always consistent with the results in vivo. For example, both of the levels of hcmv-miR-US25-1 and hcmv-miR-UL112-1 were induced in the early infection of HCMV and gradually increased as the infection progressed, which could attenuate the replication of HCMV and implicated in latency control of HCMV by targeting HCMV IE1, UL112/113, UL120/121 and UL144 [8, 9]. However, in our study, the expression of hcmv-miR-UL112-1 in patients who suffered the reactivation infection with HCMV were not significantly higher than that in latent infection patients. We speculate that hcmv-miR-UL112-1 may play a role in inhibiting viral replication in the early stages of infection but not in late stage. Furthermore, one recent study found eight highly expressed HCMV-encoded-miRNAs in latently infected cells, with hcmv-miR-UL112-3p and hcmv-miR-US22-5p were the most abundant miRNAs during latency [20], however, these two miRNAs were also not showing significant upregulation upon reactivation in our present study. Hcmv-miR-US29-5p and hcmv-miR-US29-3p were expressed at different stage of HCMV infection pattern. During lytic infection, hcmv-miR-US29-5p was expressed and undetectable during latent phase, while the opposite occurred with hcmv-miR-US29-3p [20]. This was also inconsistent with the results in this study that the level of hcmv-miR-US29-3p was significantly higher than that of latent infection after reactivation infection. Additionally, hcmv-miR-US4-5p was reported to promoted apoptosis via inhibition of p21-activated kinase 2 expression in cultured cells which may establish a balance between the host cell and virus during natural HCMV infection [27], however, whether or not this HCMV-encoded-miRNA as the regulatory molecules in order to maintain HCMV latent state in vivo and the difference expression pattern between in vivo and in vitro remains to be further verified.

Latest studies of the hcmv-miRNAs as diagnostic indicator were found in patients with glioblastoma, rheumatoid arthritis (RA), diabetes mellitus and essential hypertension by detection with circulating hcmv-miR-UL112-3p [14, 15]. Comparatively, detection of hcmv-miR-UL22A-5p in transplant recipients with HCMV infections had the highest sensitivity for the prediction of subsequent virologic recurrence [16]. Our research group also demonstrated different expression profile of HCMV-encoded miRNAs in plasma sample from patients suffered with OLP, and 5 of the miRNAs including hcmv-miR-UL112-3p, hcmv-miR-UL22a-5p, hcmv-miR-UL148D, hcmv-miR-UL36-5p and hcmv-miR-UL59 were significantly upregulated in OLP samples compared with normal samples [17]. Here, we identified highly hcmv-miRNAs expression pattern upon reactivation from latency in vivo, especially for hcmv-miR-US25-1-3p in autoimmune disease patients. Of the eight HCMV miRNAs, hcmv-miR-US25-1-3p showed the greatest diagnostic performance. To the best of our knowledge, there was no report about the relationship between HCMV-encoded miRNAs and autoimmune diseases. In warfare terms, HCMV is in a ‘stand-off’ relationship with immune system, poised to replicate rapidly if the established immune response becomes impaired, which can occur in patients given immunosuppressive drugs [2]. Increasing evidence suggests that HCMV constitute an important trigger of systemic lupus erythematosus (SLE) and can further aggravate disease progression [28]. Chronic inflammation in the autoimmune patients is a driving force for reaction if latent HCMV [29], which results in a vicious cycle. We found that HCMV-encoded-miRNAs may play an immunomodulatory role or be a potential biomarker for the switch of HCMV from latency to reactivation, which provides a key foothold for the study of HCMV virology, even though hcmv-miRNAs’ function remain to be further studied in natural diseases such as in autoimmune disease.

In our present study, we also found that the expression levels of hcmv-miR-US25-1-3p was consistent with HCMV DNA level in the HCMV antiviral therapy period of patients with lymphocytic leukemia who accepted with hematopoietic stem cell transplantation, which suggested that HCMV miRNAs expression may be efficient for the diagnosis, evaluation and prediction for HCMV infection. Notably, for patient No. 4, HCMV DNA levels was consistent with antiviral therapy, while hcmv-miR-US25-1-3p expressed earlier than the expression of HCMV DNA and decreased later than HCMV DNA during the antiviral therapy (Fig. 6d). This means that the expression of hcmv-miR-US25-1-3p in this patient may be earlier than the expression of HCMV DNA, although the HCMV DNA drops to a negative level after antiviral treatment, it may be possible that subsequent patients may trigger the appearance of viremia again.

The origination and function of HCMV miRNAs in circulation was not fully promulgated currently; however, most organs and tissues can be infected with HCMV in vivo due to the broad cell tropism of the virus [22], such as fibroblasts and smooth muscle cells are fully permissive to lytic replication [22], endothelial and epithelial cells of many organs undergoes a more protracted replication cycle that results in persistent low-level release of virus, and less differentiated CD34^+^ hematopoietic progenitor cells in the bone marrow as well as CD14^+^ monocytes being maintained latency infection [21, 30, 31]. Thus, all of the above-mentioned cell types may be the potential source of HCMV miRNAs. On the other hand, latency necessarily requires a homeostasis between host and virus, and a successful reactivation from latency under suitable conditions, such as following mobilization of stem cells from the bone marrow and myeloid differentiation into macrophages should be the full viral gene expression [22]. All these results indicate that these HCMV-encoded-miRNAs may share similar physiological and pathological roles in HCMV infection related diseases mentioned above, as well as in this study, even though much further study is needed to confirm this. In summary, these HCMV-encoded-miRNAs may be potential biomarkers for indication of the switch of HCMV from latency to reactivation.

## Conclusion

To the best of our knowledge, we are the first to report a dynamic characterization profile of HCMV encoded miRNA in circulation of HCMV infected patients from latency to reactivation. We identified that hcmv-miR-US25-1-3p may be used as a potential predictor for the switch upon reactivation from latency in patients suffered with autoimmune diseases. These findings may reveal important insights into the pathogenesis of HCMV infection.

## Supplementary Information

**Additional file 1: Table S1.** Dynamic changes of HCMV DNA and hcmv-US25-1-3p levels in the 5 patients that receiving bone marrow transplantation during antiviral therapy.

## Data Availability

The authors confirm that the data supporting the findings of this study are available within the article.

## Abbreviations

HCMV: Human cytomegalovirus
RT-qPCR: Quantitative reverse transcription polymerase chain reaction
miRNAs: MicroRNAs
qPCR: Quantitative real-time PCR
ELISA: Enzyme linked immunosorbent assay
ROC: Receiver operating characteristic curve
OLP: Oral lichen planus
AUC: Area under curve
OD: Optical density
CI: Confidence interval
RA: Rheumatoid arthritis
SLE: Systemic lupus erythematosus
ALM: Acute lymphoblastic leukemia
AML M2a: Acute myeloid leukemia M2a
