# Supplementary material for: Different expression pattern of human cytomegalovirus-encoded microRNAs in circulation from virus latency to reactivation
Source: J Transl Med. 2020 Dec 9;18:469. doi: 10.1186/s12967-020-02653-w (PMC7727131; doi:10.1186/s12967-020-02653-w)
Supplement: Supplementary file 1 — Additional file 1: Table S1. Dynamic changes of HCMV DNA and hcmv-US25-1-3p levels in the 5 patients that receiving bone marrow transplantation during antiviral therapy. [file 12967_2020_2653_MOESM1_ESM.docx]

**Additional files**

**Table S1. Dynamic changes of HCMV DNA and hcmv-US25-1-3p levels in the 5 patients that receiving bone marrow transplantation during antiviral therapy.**

| **Patient No.1** | | | **Patient No.2** | | | **Patient No.3** | | | **Patient No.4** | | | **Patient No.5** | | |
| --- | --- | --- | --- | --- | --- | --- | --- | --- | --- | --- | --- | --- | --- | --- |
| **Days ^a^** | **HCMV DNA**  **(IU/mL)** | **hcmv-miR-US25-1-3p**  **(2^-ΔCq^)** | **Days ^a^** | **HCMV DNA**  **(IU/mL)** | **hcmv-miR-US25-1-3p**  **(2^-ΔCq^)** | **Days ^a^** | **HCMV DNA**  **(IU/mL)** | **hcmv-miR-US25-1-3p**  **(2^-ΔCq^)** | **Days ^a^** | **HCMV DNA**  **(IU/mL)** | **hcmv-miR-US25-1-3p**  **(2^-ΔCq^)** | **Days ^a^** | **HCMV DNA**  **(IU/mL)** | **hcmv-miR-US25-1-3p**  **(2^-ΔCq^)** |
| 26 | < 500 | 0.0769465 | 44 | 588 | 0.0124 | 27 | 1590 | 0.02105 | 22 | < 500 | 0.004 | 26 | < 500 | 0.003 |
| 33 | 1930 | 0.0984135 | 65 | 6810 | 0.0759 | 33 | 2020 | 0.01504 | 42 | 2460 | 0.007 | 33 | 3890 | 0.0053 |
| 38 | 5290 | 0.0693481 | 72 | < 500 | 0.0094 | 63 | 4830 | 0.01971 | 49 | 1200 | 0.002 | 36 | 597 | 0.0021 |
| 47 | 526 | 0.0371627 | 79 | 13400 | 0.0079 | 83 | < 500 | 0.00452 | 56 | < 500 | 0.002 | 40 | 701 | 0.0012 |
| 49 | < 500 | 0.052556 | 92 | 1020 | 0.0105 | 97 | < 500 | 0.00262 | 63 | < 500 | 0.003 | 43 | 1280 | 0.0016 |
| 76 | < 500 | 0.0088814 | 102 | < 500 | 0.0113 | 108 | < 500 | 0.0087 | 152 | < 500 | 0.006 | 47 | 2820 | 0.0057 |
|  |  |  | 130 | < 500 | 0.0158 | 118 | < 500 | 0.00291 |  |  |  | 51 | 501 | 0.0013 |
|  |  |  | 136 | < 500 | 0.0085 | 135 | < 500 | 0.00224 |  |  |  | 62 | < 500 | 0.003 |
|  |  |  | 164 | < 500 | 0.0054 | 149 | < 500 | 0.00218 |  |  |  | 132 | < 500 | 0.001 |
|  |  |  |  |  |  | 165 | 1960 | 0.00108 |  |  |  |  |  |  |
|  |  |  |  |  |  | 169 | 9770 | 0.00694 |  |  |  |  |  |  |
|  |  |  |  |  |  | 172 | 10800 | 0.00224 |  |  |  |  |  |  |
|  |  |  |  |  |  | 181 | 1250 | 0.00489 |  |  |  |  |  |  |
|  |  |  |  |  |  | 184 | 1700 | 0.00397 |  |  |  |  |  |  |
|  |  |  |  |  |  | 188 | 6630 | 0.00632 |  |  |  |  |  |  |
|  |  |  |  |  |  | 191 | < 500 | 0.00368 |  |  |  |  |  |  |
|  |  |  |  |  |  | 200 | 577 | 0.00864 |  |  |  |  |  |  |

a, days after transplantation.
